# Supplementary material for: Patterns of Intron Gain and Loss in Fungi
Source: PLoS Biol. 2004 Nov 30;2(12):e422. doi: 10.1371/journal.pbio.0020422 (PMC532390; doi:10.1371/journal.pbio.0020422)
Supplement: Table S1 — Also available at http://genes.mit.edu/NielsenEtAl/. (4.3 MB ZIP). [file pbio.0020422.st001.zip › NielsenEtAl/html/444.html]

AN2529.1.NCU06647.1.MG07309.1.FG09979.1


```
 CLUSTAL W (1.82) Multiple Sequence Alignments - Introns Inserted


Sequence 1: NCU06647.1	290 aa
Sequence 2: FG09979.1	285 aa
Sequence 3: MG07309.1	285 aa
Sequence 4: AN2529.1	280 aa
Alignment Length: 296 aa
Number Identitical Residues: 131 aa
Alignment Score (without introns) 6421


MG07309.1 	MSSQ----------SYAHFLVHRPAP-GVAHVEINRPDKLNSFYEE~MWLEFGRVFNALS
NCU06647.1	MAQQQQQPQLKGYTSYKHFNVTSAGPAFVVHVEINRPAKLNAFFEA~MWLELGQIFRQLS
FG09979.1 	MSTATPIP---AYASYKHMLVSSPGP-FVAHVEINRPKKLNAFSQE~VWLEFGHVFNQLS
AN2529.1  	MASN---------YSPKYFNINFPQE-YVAHVEINRPNQLNAFFEA2MWLELGQLFAQLS
          	*:            *  :: :  .    *.******* :**:* :  :***:*::*  **

MG07309.1 	VDPEIRAVVLSGAGERAFTSGLDVQKASDGWLVKGMDDGSGQPVDSARFATYARRHIAEF
NCU06647.1	SDPDVRAVVLSGAGDRGFTAGLDIHAASSNNQLQSSG-----ALDVGRKAAQLRRYITEF
FG09979.1 	NDSDIRAVVLSGAGDRAFTAGLDVQAASGDG-ILSDA-----AGDVAKKAKVLRNHIEEF
AN2529.1  	TDPAVRAIVISGAGTKAFTAGLDVKAASQGLLSSDSK-----ASDPARKAVHLRREVGSF
          	 *. :**:*:**** :.**:***:: ** .    .       . * .: *   *. : .*

MG07309.1 	QDSISAMEKCEKP1VICVLHGLSLGLAIDLACCADVRLVARGTRMAVKEVDIGLAADIGT
NCU06647.1	QDSISAIELCEKP1VIVVLHAISLGLAIDIACAADIRLCTRDVRLAVKEVDIGLAADIGT
FG09979.1 	QDSISAMEKCEKP1VICVLHGIAIGLAIDIACCADIRFCASTVRFAVKEVDIGMAADIGT
AN2529.1  	QDCVSSIEKCEKP~VIVAMHGFSLGLAIDLSSAADVRFCAKDTRFAVKEVDIGLAADVGT
          	**.:*::* **** ** .:*.:::*****::..**:*: :  .*:********:***:**

MG07309.1 	LSRLPKAVGSLSWVKEVCLSAREFTPEEALRVGFVSGPIHETKADAVKAALSMAALVASK
NCU06647.1	LSRLPKAVGNFSWVKEVCMSAREFGATEAQQVGFVS-QVLDSKVAALDQAIKLAGLIAGK
FG09979.1 	LSRLPKIVGSTSWVKDVCLTARDFSAQEALSMGFVS-QVHENKQATVQAAVALATTLAEK
AN2529.1  	LSRLPKIVGNYGWVKDVALSARLFGAEEALSVGFVS-RVFETKEEAVKGAIELAALIASK
          	****** **. .***:*.::** * . **  :****  : :.*  ::. *: :*  :* *

MG07309.1 	SPVAVQGTKELLNHARDHSVEES~LRYTAVWNSAALQTRDIKDALLSGIQKRKATFAKL
NCU06647.1	SPIAVQGTKELLNHARDHSVSES1LRYTTVWNSAALQSRDMMDALSASVQKKKPVFSKL
FG09979.1 	SPVAVQGTKELLNYSRDHSVAEG~LRYTKVWNAAAVQGRDFAVGLTSGLKKTKPRFEKL
AN2529.1  	SPVAVQGTKELLNWSRDHTVQDG1LRYTAVWNSAALQTSDVQSALLSGIQKRKPTFEKL
          	**:********** :***:* :. **** ***:**:*  *.  .* :.::* *. * **
```
